# Policy processes *sans frontières*: interactions in transnational governance of global health

**DOI:** 10.1007/s11077-020-09375-2

**Published:** 2020-02-26

**Authors:** Catherine M. Jones, Carole Clavier, Louise Potvin

**Affiliations:** 1 LSE Health, London School of Economics and Political Science, London, UK; 2 Regroupement stratégique Politiques publiques et santé des populations, Réseau de recherche en santé des populations, Montréal, Québec Canada; 3 Département de science politique de l’Université du Québec à Montréal, Montréal, Québec Canada; 4 Centre de recherche en santé publique (CReSP), Université de Montréal and CIUSSS Centre-Sud-de-l’Île-de-Montréal, Montréal, Québec Canada; 5 Département de Médecine sociale et préventive, École de santé publique de l’Université de Montréal, Montréal, Québec Canada

**Keywords:** National policy on global health, Global health governance, Transnational governance, Intersectoral policy processes, Interaction, Policy learning, Elite networking, Boundary spanning

## Abstract

National policy on global health (NPGH) arenas are multisectoral governing arrangements for cooperation between health, development, and foreign affairs sectors in government policy for global health governance. To explore the relationship between national and global processes for governing global health, this paper asks: in what forms of interaction between NPGH arenas and global health governance are learning and networking processes present? In a multiple case study of Norwegian and Swiss NPGH arenas, we collected data on intersectoral policy processes from semi-structured interviews with 33 informants in 2014-2015. Adapting Real-Dato’s framework, we analyzed each case separately, producing monographs for comparing NPGH arenas. Analyzing both NPGH arenas for relational structures linking external resources to internal policy arena processes, we found five zones of interactions - including institutions, transgovernmental clubs, and connective forms. These interactions circulate ideas and soften arenas’ boundaries. We argue that NPGH is characteristic of transnational governance of global health.

**Electronic supplementary material:**

The online version of this article (10.1007/s11077-020-09375-2) contains supplementary material, which is available to authorized users.

## Introduction

The proliferation of global health actors over the past two decades has produced a dense and diverse landscape of organizations involved in global health governance. Global health governance {GHG} is a set of formal and informal processes, operating beyond state boundaries, through which actors participate in steering, coordinating, and financing collective action on health and disease in populations around the world (Dodgson et al. 2002; Lee and Kamradt-Scott 2014; Ng and Ruger 2011). As a concept, GHG is used both analytically and normatively, referring to processes for health governance in a globalized world, global institutions’ impact on health and its determinants, and arrangements for collectively establishing and meeting global health goals (Lee and Kamradt-Scott 2014). As a system, GHG comprises actors broadly categorized as public/private, state/non-state, or with old/new status, such as: UN agencies, public-private partnerships, philanthropic foundations, NGOs, development banks, think tanks, academic institutions, and governments (Youde 2012; Harman 2012; Clinton and Sridhar 2017a). However, within this evolving GHG scenery wherein non-state and private actors have gained prominence (Hoffman et al. 2015; Ng and Ruger 2011; Szlezak et al. 2010), Ricci (2009) notes that the role of the state has been underemphasized in the contemporary literature.

State actors from high-income countries (e.g. OECD Development Cooperation Committee members) are generally analyzed regarding their roles as donors (McCoy et al. 2009; Piva and Dodd 2009; Ravishankar et al. 2009). Even within the mosaic of funding from new, non-traditional donors (Schäferhoff et al. 2014; Harman and Williams 2014), 73% of official development assistance for health is from states (Moon and Omole 2017). But the state has a critical role to play as a global health governor, beyond that as a global health financier. Schrecker (2012) warns that confining our focus on complex institution building at the global level deters much needed attention from the policy processes and agency of state actors and national governments. The role of the state should not be understood as one that is diminishing relative to that of other actors in the GHG system, but rather it should be understood with regard to its interactions with other actors in the system (Dodgson et al. 2002; Ricci 2009; Davies 2010; Sandberg et al. 2016).

National policies on global health {NPGH} are public policies on global health matters involving collaboration between health and foreign affairs ministries. Switzerland’s *Swiss Health Foreign Policy* (2019, 2012, 2006) has been identified as the first example of an inter-ministerial agreement adopted on objectives for a national global health strategy (Kickbusch et al. 2007). Other examples include the U.K.’s *Health is global: UK Government Strategy* (2008), the Norwegian *White Paper on Global health in foreign and development policy* (2012), Germany’s *Shaping global health – taking joint action – embracing responsibility* (2013), Japan’s *Global Health Policy* (Okada 2010), and France’s *Strategy for Global Health* (2017). Descriptions of these global health strategy documents underscore the disposition of diplomacy and policy formulation to integrate heath and foreign policy in multisectoral arrangements for global health at the national level (Kanth et al. 2013; Hein and Kickbusch 2012; Kickbusch et al. 2007), but little is known about how these interact with processes in the GHG system at large outside of global health diplomacy instruments and practices (Ruckert et al. 2016; Kickbusch 2013). Yet, there is nascent knowledge about policy processes to develop such documents (Gagnon and Labonte 2013; Aluttis et al. 2015). A comparison of the NPGH documents adopted in Norway and Switzerland showed that these policies target actors in the GHG system (Jones et al. 2017b). NPGH arenas are multisectoral governing arrangements wherein actors from health, development, and foreign affairs sectors interact in policy action situations to make decisions about the government’s work in global health and its governance (Jones et al. 2017a). Various rules-in-use by actors for micro-processes (e.g. coordination, information sharing, and negotiation) within NPGH arenas establish ranks and relationships of power between different policy sectors interacting on behalf of the state in global health. For example, the rules in the Norwegian NPGH action arena designated a leading role to the foreign affairs sector and a supporting one to the health sector, except in matters concerning WHO affairs (see Appendix A in supplementary material). Alternatively, the Swiss NPGH arena depended on a core group of four sectors that share power in decision-making, with public health as a linking sector between them (see Appendix B in supplementary material).

As global health policy-making is carried out by actors operating at different levels of governance within a shared timeframe, the relationships between policy processes at various scales constitute elements for exploring influences on decision-making in these arenas. The problem is that these relationships may be invisible to global health policy practitioners, in particular when these relationships are built through the involvement of actors from policy sectors other than their own. This relational problem is central to polycentric governance of global health (or other policy regimes of global scope like climate change) because policy development and action at one level may not be independent of a combination of influences (cooperation or competition) from others (Tosun 2017; Gautier et al. 2018). Knowledge about potential interdependencies between global/foreign and national/domestic public policy processes pertaining to GHG may benefit policy scholars and practitioners to learn how national policy-makers from different sectors engage in GHG and where are the interfaces between GHG and national-level decision-making on state action in global health. Based on two in-depth case studies of Norwegian and Swiss NPGH arenas, this paper aims to explore the relationship between processes at national and global decision-making levels on matters of global health.

## Theoretical approach

Drawing from a review of empirical literature in health and social sciences on the relationship between global and national level policy processes, we conceptualized this relationship as top-down with the global arena influencing national arenas - in particular through the circulation of policy ideas in GHG as one explanation of policy change in NPGH (Jones et al. 2017a). Thus, our conceptual framework for this study, based on Real-Dato’s synthetic framework (Real-Dato 2009), positioned external mechanisms of policy change as a directional force from the global arena on to the national one (Jones et al. 2017a). We used literature on how ideas affect policy change to inform our selection and understanding of potential mechanisms by which the global arena influences NPGH policy arenas (Campbell 2002; Béland 2009). Mechanisms are defined as relational concepts to understand how levels of NPGH and global arenas relate (Falleti and Lynch 2009). We selected policy learning and networking as mechanisms because both suggest that the circulation of ideas and instruments between actors may explain processes for policy change and outcomes of policy change.

Policy learning is a cognitive process by which ideas are mobilized for policy change. Policy learning refers to using past and current experiences to inform ideas and policy-relevant knowledge of actors for decision-making at the collective choice level (Real-Dato 2009). Policy literature proposes various types of learning such as political, social, policy-oriented, or government learning and lesson-drawing (Bennett and Howlett 1992). Learning objectives reflect different kinds of change for doing policy: procedural (adjustments for organizational change), instrumental (lessons for programmatic change), or systemic (frameworks for paradigmatic change) (Bennett and Howlett 1992; May 1992, 1999; Howlett and Ramesh 2002; Hall 1993). This literature also distinguishes intentional from reactive learning (Bennett and Howlett 1992). For Hall (Hall 1993) learning is deliberate, by questioning previous policy successes or failures and learning from their consequences to adjust policy objectives and tools; whereas for Heclo [cited in (Howlett et al. 2009)] learning is stimulated by the social context and policy environment, which incites a process to adapt and respond to external shifts.

Networking processes among actors from different government jurisdictions, international institutions, and epistemic communities contribute to the circulation of policy ideas and instruments (Legrand 2012; Haas 1992). Elite networking often occurs within policy communities (e.g. experts inside or outside government) and between governments that share a particular issue of policy or professional interest (Bennett 1991). Elite networking also disseminates policy ideas because it connects actors with shared identities and concerns to policy learning (Bennett 1991; Stone 2001). Although networking transports ideas between structures and across levels, institutions and political context mediate whether and how ideas are considered in policy (Béland 2009; Palier et al. 2010).

Through investigating both learning and networking, we aim to identify where these mechanisms for policy change may be operating to establish a relationship between NPGH action arenas and GHG. Specifically, this paper asks: in what forms of interaction between NPGH arenas and GHG are learning and networking processes present?

## Methods

A multiple comparative case study of policy arenas prior to the adoption of NPGH in Norway and in Switzerland in 2012 was designed to answer this question, applying most similar systems design criteria for case selection (Jones et al. 2017b). Informed consent was obtained from all participants in accordance with ethical guidelines.

### Data collection

Purposive and snowball sampling was used to build a sample of relevant actors in each case as key informants. The sample was discussed and validated separately by case-specific Context Advisory Groups, each group consisting of the first and second author and one national expert on global health policy and governance for the respective case country. Based on similar studies (Gagnon and Labonte 2013; Aluttis et al. 2015), we intended to recruit approximately 15 informants per case, including actors from the foreign affairs, health and development policy sectors. Semi-structured interviews were conducted with 19 informants from Norway and 14 from Switzerland between November 2014 and October 2015 (see Table 1). We asked informants about intersectoral policy situations and decision-making processes for NPGH during the period 2006-2012 before the formal adoption of policy documents. In addition to questions about how actors worked together in policy situations, we asked about influences on processes to develop NPGH, and specifically, whether anything from outside of the respective countries exerted influence (i.e. If so, what? Where from? Who or which process contributed it? How was it used?). A diagramming technique for graphic elicitation was used in the face-to-face interviews. With one exception, interviews were recorded (36+ hours of audio) and transcribed verbatim (543 pages of transcripts). Each case was analyzed separately to produce two monographs (see summaries in supplementary material).^1^

**Table 1:** Informants classified by actor’s sphere and sector for each case

Actors’ societal spheres (policy sectors)	Key informants	
	Norwegian case	Swiss case
State actors (development-D)	3	1
State actors (health-H)	7	5
State actors (foreign affairs-FA)	4	4
State actors (intellectual property/justice-IP)		2
Civil society actors (health-H)	1	1
Public actors (global health research-H)	4	
Private actors (global health research-H)		1
	19	14

### Data analysis

These monographs provided a comparable construction of the two NPGH arenas to explore the mechanisms of learning and networking for similar or different forms of interaction with GHG (Collier 1993; Boussaguet and Dupuy 2014). Data on mechanisms of policy change related to external sources of influence (e.g. institutions, ideas, instruments) on the internal policy process of NPGH. Mechanisms of policy change are non-deterministic relational concepts because they are mobile and work differently in different contexts (Falleti and Lynch 2009). Because the study defines policy context as a composite of physical environment and ideational elements, context includes the social, scientific/technical, and political fabric within which actors work. As such, contexts are elements of NPGH arenas, but they also span across boundaries of sovereign jurisdictions and geographical national borders. This has two implications for analysis: we did not analyze outcomes of mechanisms operating in the cases, and we looked for mechanisms of learning and networking in contexts that were relevant from informants’ perspectives for understanding their government’s work in global health, whether inside or outside the national arena.

According to Hassenteufel and Palier (2001), relational analysis seeks to connect the internal {national} and external {global} levels rather than place them in opposition with one another. Informed by their distinction between unilateral (seeking to understand how external factors impact NPGH arenas) and transnational (breaking with internal/external as opposing categories to understand the interactions between them) analyses (Hassenteufel and Palier 2001), we searched our data for the relational structures that appeared to forge a connection between NPGH arenas and GHG. We use an interpretive schema of interaction to explore the zones that connected national and global level actors where learning or networking mechanisms operate. We examined the resources (e.g. knowledge, programs, networks, frameworks) from outside the NPGH arenas reflecting on their provenance, their proponents, and their prioritization. We also recognized learning when resources were linked to experiences and lessons for modifying policy practices. We highlighted data pointing to significant international meetings, institutions, initiatives, and partnerships in which informants signaled participation of actors from their NPGH arena.

## Results

Analyzing two NPGH arenas (hereafter referred to as Norwegian arena or Swiss arena), we found five forms [F1-F5] of interaction between NPGH processes and the international context wherein mechanisms of policy learning and elite networking operate.

### F1 Governing bodies of intergovernmental institutions for health

We found policy learning processes operating in the interactions between the Swiss and Norwegian arenas and GHG when countries participated in the governance of international institutions responsible for health, namely the World Health Organization {WHO}. The World Health Assembly {WHA} and the WHO Executive Board {EB} were zones of interaction in both cases, appearing linked to reflexive approaches of member states to reorganize their participation in WHO’s governing bodies. During the 2000s, the politicization of the WHA stimulated learning for organizational change to include more senior politicians for authority in delegations. From WHA interactions, the NPGH arenas learned that their WHO delegates needed more diplomatic skills for negotiating, in addition to technical skills related to health and development.

There was this idea that it’s an either/or thing. It’s either foreign policy or health policy. Which in the case of WHO is no longer correct. The two spheres have really been blurred… and it’s crucial that our two ministries work very closely together. It is much more politically driven. *Norwegian informant (H)*

Learning processes in the WHA prompted government innovations in foreign affairs administration, like health expertise for diplomatic posts and WHO liaison staff in permanent missions. In the Swiss arena, learning supported the creation of a health sector desk in the political division of the Federal Department of Foreign Affairs, nomination of health-dedicated personnel in the Permanent Mission of Switzerland to Geneva, development of health diplomacy training for health attachés in embassies, and establishment of Ambassador for Global Health title.

When I started, the missions in Geneva had no one specifically trained in or assigned to health. Now, that is almost standard. *Swiss informant (H)*

In the Norwegian arena, the organization of the Permanent Mission of Norway to Geneva reflects intersectoral cooperation on health with diplomatic councilors for WHO matters from both health and foreign affairs ministries.

The Swiss arena experience showed that without “any [interdepartmental] governance process and coherence procedure, you may not properly defend your interests on the international level.” *Swiss informant (IP)* The ideas for inter-ministerial cooperation instruments in the Swiss arena were fostered by Swiss actors’ deliberate learning from the conflicts between sectors in WHO governing bodies.

The starting point of the *Swiss Health Foreign Policy*, and the really disturbing point was particularly in the context of the WHA, that representatives of different branches of the Swiss government had very different views on things. It really crystallized around the question of Nestle and baby food. *Swiss informant (H)*

The interactions in matters of WHO governance constituted learning processes for the Swiss and Norwegian arenas to reflect on organization and representation of the sectoral dimensions of cooperation in the delegations, and to establish rules for preparing, taking, and administering WHA decisions between sectors that were necessary to manage onsite demands during the assembly. The NPGH arenas in both cases held multisectoral information meetings before each WHA and EB meeting in addition to specific delegation meetings.

We have the WHO Forum prior to the WHA in May, the EB in January, and the European Regional Committee in September. We go through the main topics for these WHO meetings and agree on which agency takes responsibility to develop issue papers. We go very strategically through the agenda, prioritizing items, so people going to the meetings are well prepared when it comes to the topic’s background and questions, and with the Norwegian position. *Norwegian informant (H)*

Similarly, in the Swiss arena, learning in this interactive space triggered processes for defining united positions in advance, rather than assembling a collection of sectoral ones. This represents a major shift from the preparatory processes for WHA before the coordinated approach was institutionalized through the Swiss arena.

We did not even have a preparatory meeting in Bern before the WHA. We just went to Geneva and the different parts of the delegation met in the hall before the assembly started. And then, sometimes we realized that we had major issues where we disagreed. That was very difficult because someone had to call the Ministry of Foreign Affairs about what to do. *Swiss informant (H)*

From the Swiss arena’s perspective, the WHO governing bodies are interactive spaces for policy-oriented learning because governing bodies validate and circulate global norms for national adaptation. As one *Swiss informant (FA)* said, “the WHO agenda strongly influences the priority topics” deliberated in the Swiss arena. The agendas of formal meetings of interdepartmental groups also included topics from international organizations for which health is not the focus, such as World Intellectual Property Organization.

In addition to the annual WHA, the biannual EB generated frequent interactions between NPGH arenas with a smaller group of representatives from 34 member states and the WHO secretariat. Norway and Switzerland had representatives serving terms on the EB between 2010-2013 and 2011-2014, respectively. The EB gave NPGH arenas a unique platform in addition to the WHA to showcase their contributions and priorities and to connect with actors with similar interests and ideas.

The Norwegian arena established a WHO strategy group in 2008 to prepare joint health and foreign policy objectives and working methods for its EB term. The Norwegian term increased visibility of the Norwegian arena’s work to international actors. The Norwegian arena strategically used the 65^th^ WHA in May 2012 to announce its official policy document (*White Paper on Global health in foreign and development policy*), having consulted with select partners about its content during the EB and WHA meetings of 2011. The 2012 session was the last WHA held during Norway’s EB term, and it coincided with Margaret Chan’s appointment for a second term as WHO Director General following her nomination by the EB. As a form of interaction, the EB supplied opportunities for strengthening networks with allies, and for embedding social learning through the Norwegian arena’s promotion of its foreign policy approach to GHG in WHO.

The magnitude of that White Paper’s adoption by the Parliament became clear to us throughout the process, referring to why we considered Norway a proper member of WHO because of our massive global health effort and pointing to where we wanted to move forward. We said, “This is the forum, we need to backup the head of the WHO in saying that global health is actually about foreign policy.” Norway has to be there. *Norwegian informant (FA)*

### F2 Governance of global public-private health partnerships

Norway’s involvement in establishing global health initiatives and its representation on their governing boards constituted a significant form of interaction between its NPGH arena and the international context, which was not mentioned in Swiss data. Global health initiatives use public-private partnership models to advocate, fund, and/or implement interventions for disease-specific programs. Networking of political and knowledge elites in international organizations was formative for their interactions in public-private partnerships for health where learning increased political capital in the Norwegian arena.

Jonas Gahr Støre, who later became Minister of Foreign Affairs, was Gro H. Brundtland’s right hand in WHO. At the same time Jens Stoltenberg’s appointment to GAVI’s board (2001-2005) was facilitated. So when Stoltenberg became the Prime Minister (again in 2005), he had been a GAVI board member, bringing that sphere with him and all the low-hanging fruits of success from putting money into vaccines for saving children. *Norwegian informant (FA)*

The involvement of Norwegian politicians in the governance of global health initiatives like GAVI connected policy learning directly to the political context of the Norwegian arena. Using elite networking to help place Norwegian politicians (i.e. Jens Stoltenberg and Dagfinn Høybråten) into such roles also supported policy learning useful for them in Norway. Høybråten, elected as a Board member of GAVI in 2006 (replacing Stoltenberg who became Prime Minister in 2005), was Chair of the GAVI Board from 2011-2015 during which time he was also a member of the Parliament’s Foreign Affairs and Defense Standing Committee from 2006-2013 and its spokesperson for the public hearing and committee’s opinion on the White Paper in the Norwegian arena.

Høybråten played a crucial role in ensuring a cross-spectrum support in the Parliament for this White Paper, but in doing that, he also had a lot of power in ensuring that perspectives other than those initially presented in the White Paper became substantial. *Norwegian informant (CS)*

The complex relationships of the Norwegian arena to interactive zones in the governance of institutions such as the Global Fund, UNAIDS, GAVI were intensified due to Norway’s role in their establishment. New forms of interaction with the international context of global health sprouted from the Norwegian arena’s direct and intentional modification of the GHG institutional landscape.

It’s circular because we were a big actor in setting up those funds. From that perspective, they were partly created as tools for our political priorities. We didn’t just orient towards them after they existed. And now that they are there and are doing well and giving good results, they are still our priorities. *Norwegian informant (FA)*

### F3 Transgovernmental clubs

Both arenas used formal and informal transgovernmental arrangements as interactive forms for learning. An annual retreat hosted by the Swiss Federal Office of Public Health (FOPH) in Glion prior to the WHA (2004-2011) constituted the most informal form of interaction combining elite networking and policy learning. The Head of International Affairs at the FOPH initiated this meeting between senior international affairs administrators from Ministries of Health and health experts from OECD countries to facilitate exchanges of policy ideas and experiences, creating a space for policy-oriented learning about GHG between health actors from high-income countries. For example, the idea for a WHO Committee C proposal emerged from Chatham House rules discussions in Glion.

On an informal basis, in terms of the issues [for the *Swiss Health Foreign Policy*] - a lot came out of discussions with a selection of OECD countries assembling in this little village. Some said, “It doesn’t work yet,” or, “We would like to have such a thing, but what’s your experience with that?” We found some had a paper, but not many tools. Some had tools of collaboration in place, but they hadn’t [officially] formalized it as a government decision. *Swiss informant (H)*

The Norwegian Ministry of Foreign Affairs {MFA} founded the Foreign Policy and Global Health {FPGH} initiative, a more formal diplomatic arrangement for cooperating with ministries of foreign affairs in France, Senegal, Brazil, Indonesia, South Africa, and Thailand. The FPGH initiative functioned as an interactive zone between the Norwegian arena and MFA counterparts to collectively reflect on health from a foreign policy perspective. As put by a *Norwegian informant (FA),* it was “a turf on its own that we used when we saw that it was smart … which is as important as pouring money on specific things.” The FPGH espoused a “platform of trust”, a protected space for dialogue among peers in countries from northern and southern hemispheres. By networking with actors who were not based in health ministries, it created opportunities to work “flexibility and strategically” on health in the foreign affairs policy community.

[T]he design with these seven countries across regions and alliances was unique, as an initiative without a permanent secretariat and no website. It was based on people, trust, and mutual interests. … People could come together, air disagreement and have discussions. It was a really good place to talk about definition of concepts and issues like global health security. *Norwegian informant (H)*

Policy learning from this FPGH initiative enhanced the Norwegian arena’s understanding of health issues in foreign policy terms, and this learning supported action in institutions for GHG. One policy-oriented learning outcome of the FPGH was an annual UN General Assembly resolution on FPGH and reports to the UN Secretary General which concretized commitments taken among the group of seven and increased their visibility on a global platform. The group selected a topic annually for focus in these global statements, and FPGH members transferred them to their domestic policy contexts for the MFA to reflect them in their own foreign policy agendas.

That involved connecting health to different parts of the MFA. This year, the focus for the UN General Assembly resolution has been security of health workers, so we work really closely with the humanitarian section, which also has this as a priority. And if it was environment and climate, as it was one year, we do it together with our colleagues in the climate section. *Norwegian informant (FA)*

The most formal transgovernmental interactions of the Swiss arena with the international context took the form of bilateral arrangements. Swiss NPGH actors had frequent bilateral discussions with countries that were developing similar national strategies or experimenting with health diplomacy instruments related to GHG. Bilateral relationships with countries that shared approaches and values for global health policy provided opportunities for regular meetings to share lessons learned and examples of other’s successes and challenges.

### F4 Global health hubs

Sites like Geneva and New York function as interaction zones, wherein policy ideas circulate through elite networking processes among state and non-state actors, including scientists and private foundations. Geneva, often referred to as the ‘global health capital’, hosts headquarters of global health organizations, partnerships, and financing mechanisms. Both of these cities are hubs for interaction that provide access to global health elites and experts from international organizations, policy networks, think-tanks, or NGOs.

Geneva puts the GHG capital in the Swiss arena’s backyard. Swiss actors reflected on the embedded nature of their national arena in the global one, which means they are in a continuous networking and learning process with other actors from around the globe.

[T]he most important driver for change in this *Swiss Health Foreign Policy* setup is the practice in Geneva, where we associate with different actors for different topics. Then everything is brought together within WHO. There, we take positions, we learn if we were successful and why we were not successful, and what should be changed in the future to be more successful. The missions in Geneva organize many issues on global health governance. This is our learning field. That’s why we are also very proud to have all these actors in Geneva because this is an incredible opportunity for Switzerland to influence the global thinking on global health. That’s our playground somehow. *Swiss informant (D)*

One way the Swiss arena optimized this hub was via working lunch seminars. The Swiss Interdepartmental Working Group on Intellectual property, Innovation and Public Health holds these before its formal meetings, inviting international experts to present alternative policy options that stimulate discussion and inform decision-making of the group. These policy dialogues are forms of interaction designed for the Swiss arena to learn from actors, like Medicines Patent Pool or Drugs for Neglected Diseases Initiative, aiming to use this learning for its work with Swiss pharmaceutical companies and for developing Swiss positions on GHG agendas.

Both Geneva and New York were hubs for interaction between the Norwegian arena and GHG. Networking between Norwegian elites and private actors, like the Bill and Melinda Gates Foundation, flourished through interactions in Geneva. The networks of the small “circle” of political and knowledge elites from Norway who had worked in Geneva were invaluable to the NPGH arena, helping put issues of “public-private partnerships with the World Bank, private sector money from Gates” on the agenda. New York interactions linked the FPGH initiative to global governance at large via the UN General Assembly, but it was also an important zone of interaction where elite networking connected the national and international politics of GHG.

Look at the opening week of the General Assembly over the last years. It started to become a parade ground for world leaders to show their commitment to global health. They largely do that with promising money or appearing together with prime ministers from elsewhere. That’s where *Every Woman, Every Child* came in. Norway was very instrumental in creating some of these. *Norwegian informant (FA)*

### F5 Boundary spanning transnational communities

Boundary spanning refers to forms of interaction that cut across structural boundaries of organizations, professions, sectors, cultures, socio-economic contexts, and jurisdictions (Williams 2002,2012). Transnational elite boundary spanners were not participants in the main policy action situations, yet these individuals are priceless resources for both NPGH arenas because they work across sectors and scales and foster their arenas’ connections to significant transnational actors.

In the Norwegian arena, two knowledge elites were central for boundary spanning. Both were well-renowned medical doctors with experience in developing countries. Their careers of over 40 years evolved with the changes from tropical medicine, to international health, to global health as a policy field. Their work “on the frontlines” of global health practice, research, and governance contributed directly to building the international profile and presence of Norway in GHG, simultaneously constructing pillars of the Norwegian arena.

The *White Paper on Global Health* sums up many years of policy, of activities, and of networking. And in that White Paper, we managed to spell out the importance of those personal networks, but without pinpointing the two of them. *Norwegian informant (FA)*

The Norwegian arena capitalized on their personal and professional networks, knowledge and ideas. Their careers took them from working in the field in resource-poor countries to political appointments to international organizations.

He’s been very influential in terms of the direction and the impact of Norwegian global health policy – first, by virtue of his scientific approach and institutional experience, not least at the international level. Second, because he is very strategic. Not only within this field, but across fields. *Norwegian informant (D)*

Each had direct contact with ministers, and they regularly briefed senior administrators who were responsible for connecting their strategic efforts and practical considerations in the Norwegian arena.

They were building and maintaining networks and linking people working at different levels, knowing how to pull on the good people around them… that map of politicians, before they came into position and when they were in position, the political background and networking tied around these two persons. That’s a pretty important piece of that puzzle. Because without it, we wouldn’t be where we are with the White Paper now, because we wouldn’t have had that political commitment to it in this ministry. *Norwegian informant (FA)*

In the Swiss arena, one boundary spanning knowledge elite was critical for connecting Swiss state actors to the international level. She was a significant knowledge broker and partner of the Swiss arena on whom it relied for expertise in health diplomacy and foreign policy. Her personal and professional connections built through her career and years of experience in policy, academia, and international health institutions like WHO, made her an asset to the Swiss arena as a “facilitator”.

She wrote articles and had a lot of contact [with FOPH]. Apart from our steady contact with her, she was going around the world, giving conferences on global health policies and issues. We also got the feedback from that side. She was our intellectual partner. *Swiss informant (FA)*

She especially bridged the Swiss arena with epistemic and policy communities in the external context. The establishment of the Global Health Program at the Graduate Institute in Geneva constituted a fundamental networking and learning arm for the Swiss arena; many Swiss health attachés and diplomats have been educated there. The development of the health diplomacy training established the Graduate Institute as a key stakeholder of the Swiss arena for policy dialogues with international experts in Geneva about GHG issues.

## Discussion

These results show the relationship of influence between GHG {external} and NPGH {internal} levels of policy as one of interaction between international and national processes rather than a global causal force exerting influence on domestic policy arenas. Through mechanisms of learning and elite networking, these interactions constructed interdependence via feedback between the NPGH arenas and the GHG arena. These interactions are part of an apparatus of transnational governance of global health where actors learn and network, within an evolving context for collective action among them. Our findings show that they take at least five forms varying in terms of their degree of formality, the actors involved, and their ontological status (see Table 2).

**Table 2:** Characteristics of forms of interaction between NPGH arenas and GHG

Forms of interaction	Types	Actors	Ontologies	Mechanisms
	Formal/informal	State actors/non-state actors	Institutions/networks	Policy learning/elite networking
F1	Formal	State	Institutions	Learning
F2	Formal	Both	Institutions	Both
F3	Both	State	Clubs	Mostly learning
F4	Both	Both	Hubs	Both
F5	Transversal	Both	Articulations	Both

### Characteristics of the forms of interaction

Governing bodies [F1, F2] are formal forms of interaction between NPGH arenas and the international context that take place according to the conventions of the institutions being governed. Mandated with international authority on health, WHO is the most representative GHG institutional form. There is knowledge about the WHA as an interactive form (Kitamura et al. 2013; van der Rijt and Pang 2015; Eckl 2017), but little about the EB. Similar to previous studies (Gagnon and Labonte 2013; Aluttis et al. 2015), our findings suggest that learning processes related to EB terms [F1] are common in cases of global health strategies in the literature. In spite of sub-optimal governing practices in public-private partnerships for health (Buse and Harmer 2007), public-private institutional forms of interaction [F2] have been shown to offer members commensurate access to information from the secretariat and to have accountability policies for monitoring (Clinton and Sridhar 2017b; Sridhar and Woods 2013), which have been shown to be lacking in WHO (Eccleston-Turner and McArdle 2017; Clinton and Sridhar 2017a).

Non-institutional forms of interaction [F3, F4, F5] include both formal and informal types. We found that among interactions involving state actors [F1, F3], those taking place in smaller transgovernmental clubs were significant because they are established on trust between participants, and thus clubs [F3] should not be neglected among state actors as an interactive form with implications for GHG. Research has previously highlighted formal diplomatic clubs (e.g. G7/8, BRICS bloc) as forms of interaction between national and international spheres with opportunities to exchange learning for GHG between members (Kirton et al. 2007; Harmer et al. 2013; Harmer and Buse 2014). In contrast to formal arrangements between governments, literature on informal ones [F3] is scarce. Sandberg and colleagues (2016) found the FPGH quasi-formal club practiced diplomacy as “complex relationship management outside of institutions” that “revitalizes the role of states” in GHG. In the UK and German cases, similar arrangements were mentioned for developing their strategies, such as the UK’s special relationship with the USA (Gagnon and Labonte 2013), and German actors’ interactions with OECD countries during the WHA and with UN agencies in Geneva (Aluttis et al. 2015).

Interactions that involve both state and non-state actors [F2, F4, F5] are formal within institutions [F2] and mostly informal in the connective forms [F4, F5]. Connective forms [F4, F5] are ontologically different from other structures in our findings, and they are rarely discussed in these terms in GHG literature. Both cities [F4] and transnational knowledge elites [F5] were forms of interactions important for consolidating learning and networking from institutional forms [F1, F2]. Boundary spanning, a term from organizational science and administration, generally refers to practices for reaching across diverse structural divisions to benefit inter-organizational collaboration and improve complex problem management. Researchers and practitioners have argued that global health practice needs boundary-spanning approaches to work more inclusively across contexts and structures (e.g. professional, sectoral, geographical) (Sheikh et al. 2016). In our findings, we refer to boundary spanning [F5] as a form of interaction via the work of senior transnational knowledge elites whose global health careers produced extensive personal and professional networks world-wide. They are skilled networkers and strategists (not necessarily working in government), dedicated “reticulists” and “entrepreneurs of power” (Williams 2002) with vast international experience and thick address books, who broker learning and relationships between policy-makers, funders, institutions, and epistemic communities. State actors in NPGH arenas use boundary spanning transnational knowledge elites to help them analyze and navigate the complexity of the GHG system [F5]. Through meta-networking, these elites link salient learning and relationship-building from diverse interactions in translational spaces back to politicians and policy practitioners in national institutions.

The learning produced and shared for GHG and NPGH in informal interactions [F3, F4, F5] seems complementary to that produced and shared in the formal ones [F1, F2, F4] in part because our results suggest the networking mechanisms seem more kinetic in the informal kind. This may be due to the flexibility or autonomy that informality offers NPGH actors to interact with actors from different sectors within the GHG system. Our findings showed that policy learning and networking were both operating in most forms [F2-F5], although learning was the only mechanism in data related to interactions in WHO governance [F1]. The procedural and instrumental foci of learning in the WHO governing bodies was a mixture of deliberative, based on delegations’ planning, and reactive, responding to shifts in the WHO policy environment also linked to systemic perturbations of public health crises (e.g. SARS, H1N1 swine influenza). Learning and networking processes appeared more interconnected in other forms of interaction [F2-F5] especially when networking processes are used informally to improve access to experts or policy communities and expand reach of learning processes [F4, F5].

### Transnational governance of global health

Our findings support the work of McInnes et al. (2014) who empirically define GHG as a “process of change and adaptation and as an arena where actors, institutions, and ideas interact”. Interactions between state and non-state actors from different levels of policy and governance are characteristic of the transnationalization of public policy (Stone 2004). Transnationalization pertains to relationships between actors and sectors functioning outside the traditional frameworks of international relations, and it refers to frames of reference (e.g. policy regimes, networks) relating social and geographic spaces across multiple localities, diverging from ideas of national and global spaces as concentric spheres (Pries 2009). While our results focus on forms of interaction, exploring the transnational nature of NPGH arenas calls for a discussion of agents in interactions. Stone identifies three types of “transnational policy community” individuals who circulate ideas, procedures, and instruments (Stone 2008). The “internationalized public sector official” operates in institutions and networks [F1, F2, F3, F4] based on authority from their official positions within their state. The “international civil servant” works in the secretariat of international organizations and global public-private partnerships [F1, F2, F4]. The “transnational policy professionals” are policy and practice pundits (i.e. consultant, foundation officer, scientific expert, NGO executive) [F2, F4, F5]. Transnational policy professionals impact the circulation of policy learning through networks and modify the geographies of governance (Prince 2012; Marx et al. 2012). The boundary spanning transnational knowledge elites identified in our two cases [F5] are a hybrid combination of all three types of individuals in the transnational policy community for global health due to their careers. We would add to Stone’s categories that of the “transnational capital class” of corporate elites (Carroll and Carson 2003) that interact with NPGH and the GHG system in global public-private partnerships and product development partnerships (i.e. pharmaceutical industry) [F2, F4] (see (Rushton and Williams 2011; Kenworthy et al. 2016) for examples).

The implications of these findings are that governments interpret the GHG system as a socio-political arena for exchanging ideas between state and non-state actors, in addition to an institutional arena with normative functions. Transnational interactions contribute to reaffirming state actors as intrinsic to the system. States with NPGH arenas receive recognition as integral actors in the GHG system, beyond institutional membership or donor status. The intersectoral collaboration developed within NPGH arenas aims to improve state influence in the transnational arena for GHG. Indeed, networks across state institutions and the reconfiguration of responsibilities of state agencies to include governance of issues at the international level are examples of how global governance transforms internal governance arrangements (e.g. NPGH) to perform GHG as part of the domestic arena (Hameiri and Jones 2015). Based on cases of NPGH in select high-income countries, the domestic resources for such transformations to construct NPGH arenas are not likely afforded by lower-middle or low-income countries. Furthermore, actors from these countries may be excluded from informal elite networking processes in transgovernmental and connective forms of interaction unless those forms actively build capacity for equitable representation and inclusive participation supported by skills for global health diplomacy and health in foreign policy (Adisasmito et al. 2019).

### Limitations

The exclusion of other mechanisms of policy change, such as conflict expansion and venue shopping, are limits to the study’s findings regarding forms of interaction. These types of mechanisms affect change in public policy (often in agenda-setting processes) through framing strategies to modify a policy image in order to render the policy issue more appealing or meaningful to different audiences or to propose different instruments for addressing it. We excluded these mechanisms because they are generally associated with the global governance of particular health or disease issues (i.e. HIV/AIDS, pandemics, antimicrobial resistance). Related to this point, global issues networks were absent from our data and results. Such networks have been shown to be influential in advocating issues for global health policy and practice, including helping raise political priorities (Shiffman et al. 2016; McDougall 2016; Smith and Shiffman 2016). Our observations about boundary spanning networking practices that operate at the intersections of GHG hubs and the personal networks of transnational elites from national arenas might explain why such formal global health networks are absent. These networks may represent other forms of interaction between national and global processes governing global health. However, given the focus of our study on cases of the intersectoral NPGH arena, such interactions with issue networks may be either via national civil society organizations who are observers in NPGH, through sectoral arrangements specific to health institutes or subordinate agencies, or in intersectoral arrangements at the programmatic/operational level (rather than policy governance/collective choice level). The interaction and power rules-in-use for the policy action situations in each case determined the status of civil society organizations, non-governmental organizations and academics in the NPGH arenas in Norway and Switzerland (see Appendices A and B in supplementary material). We interviewed key informants mainly from government sectors in the policy arena, as they were represented and involved in multiple action situations. This focus on actors from government policy sectors may have limited critical perspectives collected from informants on the policy process.

## Conclusion

With the objective to better understand the relationship between processes on GHG at the national and global levels, this paper inquired about forms of interaction between NPGH arenas and GHG where learning and networking processes were present. The formal and informal interactions between NPGH arenas and GHG construct an interdependent relationship between the policy processes within NPGH arenas and GHG system. These ties are formed through the circulation of ideas in policy learning and networking processes between state and non-state actors in institutions and networks. Thus, NPGH arenas appear to have fuzzy boundaries as a policy process, with their borders drawn by extent and type of their interactions with the GHG system. Based on these findings, the characterization of NPGH arenas as transnational governance of global health introduces a nuanced perspective to understand intersectoral governance arrangements of states as actors of GHG.

## Electronic supplementary material

Below is the link to the electronic supplementary material.

Supplementary material 1 (DOCX 23 kb)

## Footnotes

1 The full case monographs are available in Chapter 6 (Swiss case of NPGH action arena) and Chapter 7 (Norwegian case of NPGH action arena) of Jones’ PhD dissertation (Jones 2017).
