# Supplementary material for: Policy processes sans frontières: interactions in transnational governance of global health
Source: Policy Sci. 2020 Feb 26;53(1):161–80. doi: 10.1007/s11077-020-09375-2 (PMC7093079; doi:10.1007/s11077-020-09375-2)
Supplement: Supplementary file 1 — Supplementary material 1 (DOCX 23 kb) [file 11077_2020_9375_MOESM1_ESM.docx]

**Appendix A: Case of the Norwegian action arena for national policy on global health**

The Norwegian government occupies a niche role in the global health governance (GHG) system as a state actor instrumental in starting and supporting numerous global health initiatives. The Norwegian government’s budget and spending on official development assistance for health necessitated accountability to Parliament for its funding of diverse multilateral and public-private mechanisms for global health since the year 2000. This incentivised the development of the Norwegian arena for national policy on global health (NPGH) to rationalise global health work managed by the Ministry of Foreign Affairs within an intersectoral policy framework. The Norwegian Parliament’s formal adoption of the *White Paper on Global health in foreign and development policy* in 2012 demonstrated the Norwegian government’s fiscal responsibility to Parliament, symbolized its global health aid legacy, and validated its key role as a state actor in the GHG system. The Norwegian arena was mainly concerned with Norway’s influence on GHG, from a foreign affairs perspective, for collective action with external partners to use development aid to prevent and treat infectious diseases, and specifically to improve the health of women and children.

Between 2005 and 2013, the Norwegian arena was comprised of six main action situations [Policy Writing Group, Civil Society Organisation consultation, Public hearing, Ministerial Forum, Follow-up process, WHO Executive Board Strategy Group]. These operated under rules made by the foreign affairs sector, which gave it positional power for the leadership, coordination, and management roles in the Norwegian arena, with the health sector in a supporting role, except in matters concerning governance of WHO affairs. WHO is the only institution related to global health for which institutional arrangements were in place for coordination and joint responsibilities between the Ministry of Foreign Affairs and the Ministry of Health and Care Services. Actors in the arena formulated policy content that was technically sound and politically acceptable, interpreting various inputs and connecting them to existing practices within the foreign affairs sector bureaucracy, which housed the core team.

The rules structuring work between sectors in policy situations within the Norwegian arena appeared to reinforce a divide between sectoral cultures (e.g. ideas, approaches, instruments) of actors from the health and foreign ministries, which seemed to dichotomize rather than integrate their respective contributions to GHG. This ideational border between the health and foreign affairs sectors marked respective territories of global health claimed by each sector. Informants from the health sector perceived the Norwegian arena to be dominated by development assistance for health and disease prevention and treatment (based on an international health model), to the neglect of approaches to global health policy that promoted action on range of interdependent determinants (social, economic, political, and commercial) of health on a global scale. It was a challenge for the health sector to argue for health issues such as mental health and other non-communicable disease to be included in NPGH design because they were not part of the traditional framework of development aid for global health. Also, rules limited the relational power of non-state actors (i.e. professional associations, NGOs, researchers) making them outsiders to the decision-making processes even though their resources were valuable to government actors in the Norwegian arena. Senior politicians and scientists were key influential individuals within the Norwegian arena, even when they were not participants in action situations.

The interactions of actors from health, development, and foreign affairs sectors in the Norwegian arena generated tensions, connections, and reflections – which in turn for actors reinforced links between the ministries of health and foreign affairs, facilitated communication, and fostered better understanding of each sector’s issues, values, and strategies for global health. Through regular and mainly informal discussions, the actors from different policy sectors in the arena learned more about each sector’s policy ideas, instruments, and approaches to global health. This seemed particularly important from the perspective of health sector actors in order to build longer-term capacity for intersectoral collaboration around global health policy due to high mobility in foreign affairs sector where individuals rotate positions every few years. Although the main policy situations observed in the period of study are now inactive, actors reported that on-going dialogue between the two ministries as well as informal and ad-hoc interactions between sectors in the Norwegian arena persists (although with weak formal institutionalisation) to cultivate a sustained role for Norway as an influential state actor in GHG.

From a critical perspective, the division of responsibilities for global health and the sectoral territorialisation supported by the rules of the Norwegian arena left a number of GHG issues without policy attention or action in either health or foreign policy sectors. The sectoral division of responsibilities for health (of populations in the global south) in the Ministry of Foreign Affairs and for health (of populations in Norway and circumpolar regions) in the Ministry of Health and Care Services presented challenges for coherent policy and governance with policy-makers using different models (development aid/public health) and instruments. According to researchers, the Norwegian arena’s interactions with GHG also influenced the content of call for proposals for global health funding (i.e. GLOBVAC) earmarked in line with programme areas funded by the Ministry of Foreign Affairs, rather than concerns in line with community health, primary health care, and health policy and systems in low- and lower-middle-income countries. As such, the power asymmetry between these two sectors in the NPGH arena also impacted the Norwegian scientific and technical context.

**Appendix B: Case of the Swiss action arena for national policy on global health**

In 2005, policy actors in Switzerland began experimenting with instruments for interdepartmental cooperation on foreign policy matters concerning global health and its governance. The Federal Office of Public Health of the Federal Department of Home Affairs and the Political Directorate V of the Federal Department of Foreign Affairs developed the first interdepartmental agreement on health foreign policy objectives in 2006. In 2012, Federal Council formally adopted the *Swiss Health Foreign Policy.* The Swiss arena for national policy on global health (NPGH) resulted from ‘bottom-up’ arrangements developed by senior civil servants from health, foreign affairs, justice, and development sectors for joint decision-making on Switzerland’s action in the global health governance (GHG) system.

The Swiss arena for NPGH was constructed to make Switzerland a more credible and powerful state actor in the GHG system. Its underlying rationale rests on a core belief that collaboration between federal departments and their offices responsible for health, development, foreign affairs, and intellectual property improves Swiss decision-making processes related to GHG, and facilitates better outputs and coherent Swiss positions for international negotiations. The Swiss government aimed to improve its influence as a state actor in the GHG system by reducing contradictory positions between actors from different policy sectors representing Switzerland in global health policy venues and institutions.

Between 2005 and 2013, five main multisectoral policy situations comprised the Swiss NPGH arena. They were stratified in a hierarchy of authority so actors collaborated with counterparts in other departments at comparable levels of seniority in the administration. A political action situation at the top [Interdepartmental Conference on Health and Foreign Policy] set the political vision and institutional arrangements for monitoring at the agency director and state secretary level. A strategic action situation at the senior level [Executive Support Group] managed the strategic direction and governance of the administrative-technical situations. Administrative-technical action situations at the bottom [two Interdepartmental Working Groups: one on health and foreign policy; and one on intellectual property, innovation, and public health] coordinated the arena’s overall operations working across executive, advisory, and technical officer levels. The two administrative-technical situations were cornerstones of the Swiss arena due to the concentration of planning, policy advice, and dialogue that took place in the interdepartmental working groups between middle-senior level actors from different sectors. Although the health sector was instrumental in establishing the administrative-technical action situations, the leadership and responsibility for them were shared between a core group of health, foreign affairs, development, and intellectual property/justice policy sectors. A practice-technical action situation [Stakeholder Platform] functioned outside the public administration hierarchy with no direct influence on decisions made within the state-actor-centric situations of authority. It operated as a forum within the arena for information-exchange and networking between state actors involved with the *Swiss Health Foreign Policy* and non-state actors such as global health practitioners, researchers, educators, professionals, and industry representatives.

The position rules in the Swiss arena supported power sharing through the use of rotating chairs between actors from the sectors in the core group in charge of formal meetings. Micro-level instruments, like secondments, were also used for relational power adjustments through resource exchanges, especially between the health and foreign affairs sectors. The interaction rules for the Swiss arena required structured discussions about agenda items based on materials curated by the core group. More specifically, the decision-making rules for the Swiss arena stipulated consensus, which meant that any decision must have large majority support. Sometimes these rules produced unintended consequences according to some informants. For example, wide inclusivity may lead to consensus agreement, but on a weaker position. Also, the compulsory duty to participate and the criteria of transparency in consultations on proposals in action situations invites disagreement that must be negotiated to find compromise before making a final decision, which can slow down processes on urgent matters.

Interactions between the actors from different sectors in the Swiss arena produced four outcomes for interdepartmental collaboration – routines, models, trust, and partnerships. Institutionalising the cross-government monitoring at a high political level supported formal and informal intersectoral collaboration at the lower levels of public administration to organise, manage, and implement processes. The learning from this institutionalisation was useful for the Federal Department of Foreign Affairs to show other sectors a working model of interdepartmental cooperation on foreign policy matters. The interaction rules for dialogue and transparency, in particular in the strategic and administrative-technical situations, contributed to building relationships of trust between actors, and a better appreciation of other federal departments’ issues, interests, and instruments. The Swiss arena also created resources through decisions about new partnerships among Swiss and external actors that increased the relational power of some actors who were not a part of the core group.

The analysis of the case of the Swiss NPGH arena raises three areas of critique about the policy’s content, the arena’s rules, and the policy goal of coherence. Those actors “outside” of the decision-making action situations (e.g. academics, NGOs) critiqued the policy’s content for its lack of long-term vision, anticipation of future challenges, and operational orientation for implementing agencies. From a Swiss research perspective, the policy’s lack of alignment with *Swiss Programme for Research on Global Issues for Development* (r4d 2012-2022) was a missed opportunity for Swiss-funded global health research and knowledge development. Some actors “inside” the decision-making situations critiqued the interaction rules which were cumbersome procedural routes to consensus, to the neglect of time for interactions between sectors that could have fostered reflective, strategic, creative, future-oriented, and vision-building discussions. The goal of coherence in the Swiss arena was interpreted in a variety of ways by actors – referring to the coherence of what: process, objectives, resources. The multidimensionality of coherence as a policy goal necessitates a clear definition of its object for participants and observers to assess.
